# Evaluation of metastatic niches in distant organs after surgical removal of tumor-bearing lymph nodes

**DOI:** 10.1186/s12885-018-4538-8

**Published:** 2018-05-30

**Authors:** Jinhua Zheng, Limin Jia, Shiro Mori, Tetsuya Kodama

**Affiliations:** 1 Laboratory of Biomedical Engineering for Cancer, Graduate School of Biomedical Engineering, Tohoku University, 4-1 Seiryo, Aoba, Sendai, Miyagi 980-8575 Japan; 2 Department of Anatomy, Basic Medical Science College, Harbin Medical University, Harbin, 150081 China; 3 Biomedical Engineering Cancer Research Center, Graduate School of Biomedical Engineering, Tohoku University, 4-1 Seiryo, Aoba, Sendai, Miyagi 980-8575 Japan; 4 Department of Oral and Maxillofacial Surgery, Tohoku University Hospital, 1-1 Seiryo, Aoba, Sendai, Miyagi 980-8575 Japan

**Keywords:** Surgical removal, Tumor-bearing lymph node, Metastatic niche, Metastasis

## Abstract

**Background:**

Surgical removal of primary tumors can promote the incidence of tumor metastasis. However, molecular mechanisms underlying this process remain unclear.

**Methods:**

We inoculated tumor cells expressing luciferase gene  into subiliac lymph node (SiLN) of the MXH10/Mo-*lpr*/*lpr* mice. The tumor-bearing SiLNs were surgically removed at a certain period of time after inoculation.

**Results:**

In vivo bioluminescence imaging system and histological staining revealed metastasis in lung, proper axillary lymph node (PALN) and liver. The lung metastasis rate in SiLN removal groups was significantly higher than in the control group using Fisher exact test. Mann-Whitney U-test indicated that the luciferase-positive tumor cells in the lung and liver were significantly higher than in the control groups. The lung samples in SiLN removal groups had strong expression of lysine oxidase (LOX). Moreover, the number of CD11b^+^ cells in the lung and liver in the SiLN removal groups was significantly increased, which was positively correlated with LOX expression level. In addition, the condition of LOX and CD11b in liver was similar to lung. In the SiLN surgical removal groups, the matrix metalloproteinase (MMP)-2 and VEGFA expression in the lung tissues was significantly higher than in the control groups; the collagen fibers per area around the pulmonary vessels was quite significantly lower and negatively correlated with the expression of MMP-2 by Spearman’s analysis. Our data indicated that the reticular fibers were deposited and disordered in the tumor tissues of the lungs in the removal groups, and the reticular fibers per area was higher than in the control groups. The tumor cells in the PALN of control groups were significantly higher than in the SiLN removal groups, and CD169^+^ and CD11c^+^ cells were also higher than in the SiLN removal groups.

**Conclusions:**

Altogether, surgical removal of the tumor-bearing lymph node promoted tumor metastasis through changing the niche in lung and liver. Treatment targeting the metastatic niche might be an effective strategy to prevent tumor metastasis, thereby possibly increasing the survival and reducing the incidence of metastasis in cancer patients.

## Background

Surgical removal, radiotherapy, or angiogenesis inhibitor treatments usually have physiological impacts or induce local trauma on the body, thereby leaving a chance for the survival of tumor cells [1–3]. As a result, the harm from the tumor has been transformed from the harm caused by the primary tumor to the damage derived from metastases [4] or the harm caused by the treatment itself, but there are no effective prevention and treatment measures in clinical practice. The current detailed mechanism of tumor metastasis is not very thorough, and the therapeutic regimens for metastatic tumors are very rare and inefficient. Therefore, the clarification of the mechanism of tumor metastasis is important for the prevention and treatment of metastatic cancer.

The “seed and soil” hypothesis has long been used to explain tumor metastasis, i.e., cancer cells are considered to be seeds that spread to certain places in the body and soil refers to cytokine formation and the recruitment of cells to cultivate the proper tumor microenvironment; tumor cells eventually begin to plant and grow at the pre-metastatic site at the appropriate time [5]. Kaplan and his colleagues first proposed the concept of the pre-metastatic niche in 2005 [6]. The primary tumor cells carefully plan the formation of the pre-metastatic niche by secreting a variety of cytokines and growth factors that promote the movement of bone marrow-derived cells (BMDCs) to gather in pre-metastatic sites. Research of the pre-metastatic niche has become more and more in-depth and it has been taken into consideration that primary tumors affect and change the microenvironment of the secondary organs by promoting the formation of the pre-metastatic niche before the dissemination of tumor cells [7, 8]. Metastasis is closely associated with the formation of the pre-metastatic niche [7, 8]. A study by Kaplan et al. showed that vascular endothelial growth factor (VEGF) generated by primary tumor promotes the production of fibronectin and the secretion of MMP-9 to establish the pre-metastatic niche for invasive tumor cells [2]. Other studies have shown that primary tumors form tumor-derived secreted factors (TDSFs) to promote the recruitment of BMDCs, which react with the extracellular matrix of the target organ at the pre-metastatic site to change the microenvironment of the target organs and to promote tumor cell colonization and growth, thereby greatly enhancing the rate of tumor metastasis [6–8].

Therefore, it is necessary to further understand existing anti-cancer therapeutic techniques and tumor metastasis patterns to improve therapeutic regimens in an alternative manner, with the intention of reversing the impact of the metastatic niche and enhancing therapeutic efficacies and improving cancer prognoses.

Although the dissection of the primary tumor is beneficial, it may disturb metastatic homeostasis [9], resulting in the activation and rapid growth of latent tumors in distant organ, which has been suggested in several cancer types including breast, lung and head and neck cancers [10–12]. Due to being inspired by above papers and a model that has been proposed to explain the recurrence of breast cancer in a patient after surgical resection of the tumor [13], we wonder if removal of tumor-bearing lymph node (LN) may accelerate the occurrence of cancer metastasis. Similarly, our research showed that iatrogenic induction of distant cancer metastasis in the lung is activated after resection of a tumor-positive lymph node in a lymph node metastasis mouse model [14]. However, the concrete and detailed mechanisms are not clear.

In this study, we inoculated tumor cells directly into the subiliac lymph node (SiLN) to construct a lymph node metastasis mouse model and simulate tumor metastasis to local lymph node. This in vivo metastasis model is stable, highly efficient, and reproducible [14]. Unlike the research background of previous studies in metastasis, we did not use any in vivo models with spontaneous metastasis, but we used an in vivo model with accelerated metastasis after surgical removal of tumors.

This study examined the colonization and growth of cancer cells in the proper axillary lymph node (PALN), lung and liver tissues after the surgical removal of tumor-bearing SiLN. We also monitored the expression level of tumor cell-secreted LOX, MMP-2 and VEGFA, and the bone marrow-derived CD11b^+^ cell number in the distant organs in the in vivo model. In addition, we analyzed the expression of collagen fibers and reticular fibers in the lung of different experimental groups to evaluate if the changes of fibrous structures affect the colonization and clonal growth of metastatic tumor cells at the metastatic sites.

This study was based on the molecular changes associated with the formation of the metastatic niche after the surgical removal of tumor-bearing lymph node; we studied the mechanism of surgery-induced tumor metastasis and hope to provide new ideas and a theoretical basis to prevent and treat tumor metastasis. Further understanding of metastatic niche formation will help with the discovery of new therapeutics and lead to the radical treatment of tumor metastasis.

## Methods

Experiments were carried out in accordance with published guidelines and were approved by the Institutional Animal Care and Use Committee of Tohoku University.

### Mice

MXH10/Mo-*lpr*/*lpr* (MXH10/Mo/lpr) mice (13–15 weeks of age, 37 ± 2 g) were bred under pathogen-free conditions in the Animal Research Institute, Tohoku University [15]. The mice in the FM3A group are female, while the mice in the KM group are male.

### Cell lines

Two types of cells lines were used: malignant fibrous histiocytoma-like KM-Luc/GFP cells and C3H/He mouse mammary carcinoma cells (FM3A-Luc), which were stably expressing the luciferase gene [16].

### Lymph node metastasis mouse model

KM-Luc/GFP (final concentration: 3.3 × 10^5^ cells/mL) or FM3A-luc (final concentration: 3.3× 10^6^cells/mL) cells were suspended in a mixture of 20 mL phosphate buffered saline (PBS) and 40 mL of 400 mg/mL Matrigel (Collaborative Biomedical Products). The concrete procedures of injecting tumor cells into the SiLN and resection of the SiLN see literature 14. 3 and 6 days after inoculation of KM-Luc/GFP cells or 3 and 7 days after inoculation of FM3A-Luc cells into SiLN, the tumor-bearing SiLN was surgically removed.

### In vivo bioluminescence imaging system

Tumor development in the SiLN and metastasis to the PALN, lungs and livers were detected using an in vivo bioluminescence imaging system (IVIS; Xenogen, USA) [17]. This procedure was carried out separately at 6 h, 3 days, 6 days and 9 days after inoculation of KM-Luc/GFP cells into the SiLN (KM group), and at 3 days, 7 days, 14 days and 21 days after inoculation of FM3A-Luc cells into the SiLN (FM3A group). Moreover, the IVIS  was done immediately after and before surgical removal of the SiLN. The luciferase activities of the removed lungs, PALN and liver were measured ex vivo by IVIS on day 9 in KM group and day 21 in FM3A group, respectively.

### Tissue preparation

Mice were anesthetized using an inhaled mixture of 2% isoflurane and oxygen. All the harvested samples including lung, PALN and liver were fixed overnight in 18.5% formaldehyde, dehydrated and embedded in paraffin. Half of PALNs were embedded in OCT.

### Immunohistochemistry

Paraffin samples were sectioned at 3 μm thickness. 0.01 M citrate buffer solution (pH 6.0) was used for retrieval treatment at 120 °C for 5 min. Dako Target Retrieval Solution (S1699, Dako) was used during F4/80 immunohistochemical staining. After washing in phosphate-buffered saline (PBS), the tissue sections were incubated with normal animal serum (1:10 in PBS) from which the second antibodies were obtained for 30 min at 37 °C. A blocking kit (414,321, Histofine Company, Japan) was used according the instruction book during MMP-2 staining. Afterwards, the tissue sections were washed and incubated with the following primary antibodies at 4 °C overnight: Rabbit polyclonal antibody to Firefly luciferase (ab21176, 1:500, abcam), Rabbit polyclonal antibody to LOX (ab31238, 1:100, abcam), Rat anti-mouse CD11b Monoclonal Antibody (MCA711G, 1:500, AbD serotec), mouse monoclonal to MMP-2 (ab86607, 1:200, abcam), Rabbit polyclonal to VEGFA (ab183100, 1:50, abcam), rat monoclonal antibody to F4/80 (ab6640, 1:100, abcam), Armenian hamster monoclonal to CD11c (ab33483, 1:500, abcam). After washing in PBS, the sections were treated with 0.3% hydrogen peroxide in methanol for 20 min at room temperature (RT) to eliminate the endogenous peroxidase activity. The corresponding peroxidase-conjugated second antibodies (immediately used, Histofine company, Japan) were applied for 30 min at RT. For CD11c staining, biotin-labelled secondary antibody (ab5744, 1:500, abcam) and peroxidase-conjugated streptavidin were applied for 30 min at 37 °C. Positive reactions were developed with diaminobenzidine (DAB). The negative control was performed except that the primary antibody was replaced by PBS. Frozen sections were used for CD169 monoclonal staining. Rat anti mouse CD169 (ab53443, 1:200, abcam) was applied at RT for 2 h. The rest procedures are identical to those applied on paraffin sections.

### Special histological staining

Elastic-Masson (EM) staining combines elastic and trichrome staining techniques for demonstration and clear definition of elastic fibers of all sizes, connective tissue and nuclei in a single tissue section. Paraffin-embedded lung tissues were carried out with silver impregnation staining to display reticular fibers and to observe the distribution of reticular fibers in lungs [18].

### Computer-aided morphological analysis

Image Pro Plus 6.0 (Media Cybernetics Inc., Rockville, MD, USA) was used to calculate the intensity and extent of staining for the detected molecules, the ratio of the collagen fibers around blood vessels by EM staining and the ratio of the reticular fibers by silver impregnation staining area to the total area of the image in the lung tissues. Three microscopic fields (original magnification 200×) were randomly selected. The integral optical density (IOD) of luciferase, LOX, CD11b, MMP-2, VEGFA, CD11c, F4/80 and CD169 positive staining was calculated and was considered as the expression level of corresponding molecules. The per-area density of EM and silver positive staining was calculated to reflect the percentage of the collagen and reticular fibers.

### Statistical analysis

Statistical analyses were performed with SPSS software version 18.0 (SPSS Inc., Chicago, IL). Statistical differences were analyzed using the Mann-Whitney U-test for 2 independent groups and Fisher exact test was used to compare the incidence of metastasis (%) between groups. Spearman’s rank correlation coefficient test was used to examine the correlations among the expressions of luciferase, collagen fiber, MMP-2, Lox and CD11b. Continuous data were presented as the median (IQR). Statistical differences were considered significant when the *P* < 0.05.

## Results

### In vivo and ex vivo detection of metastases using IVIS

**Fig. 1:**
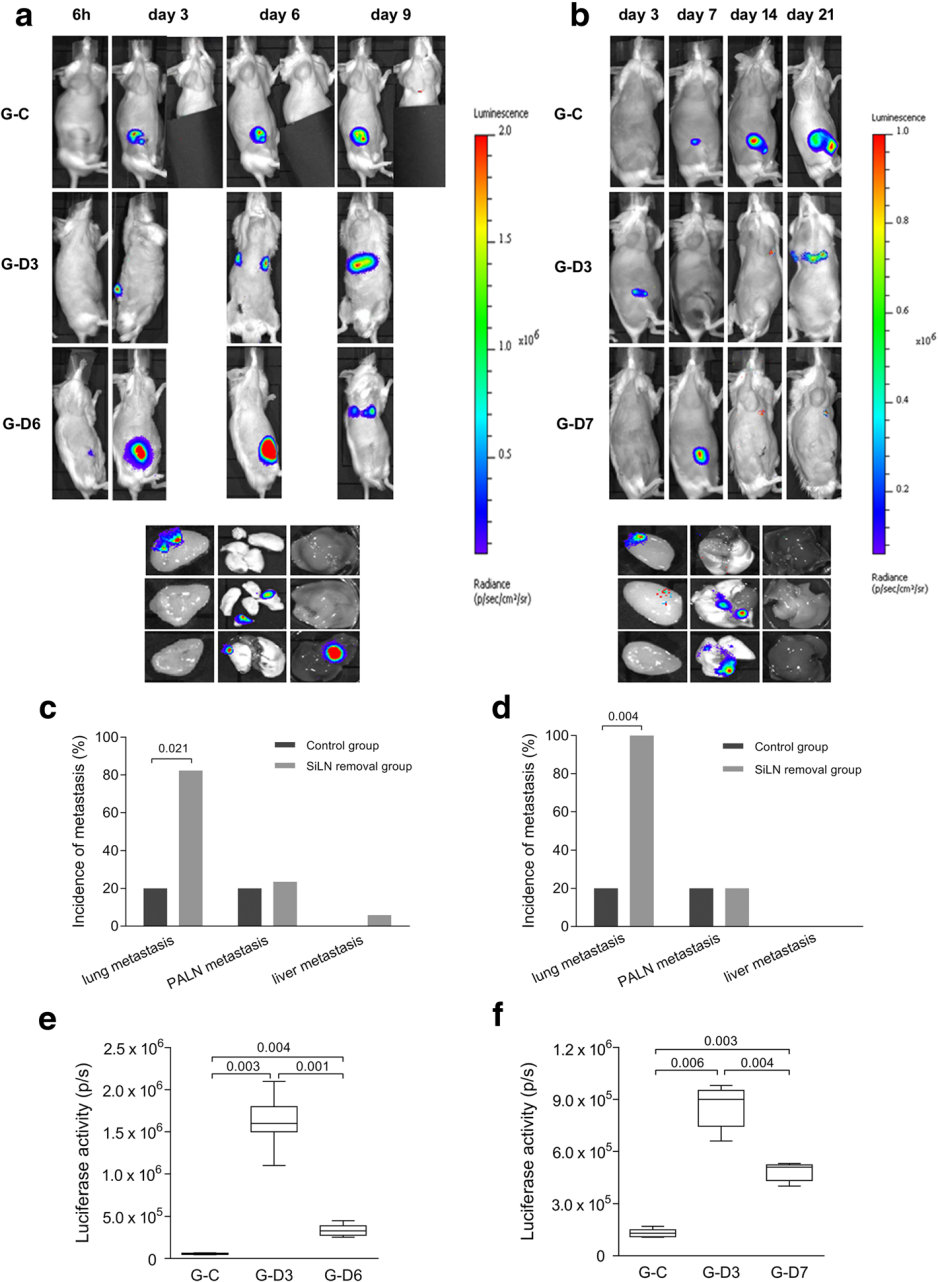
In vivo and ex vivo detection of metastases using in vivo bioluminescence imaging system. **a** KM-Luc/GFP cells were injected into the SiLN (*n* = 22) (KM group). The SiLN was removed 3 days (G-D3 group, *n* = 9), or 6 days (G-D6 group, *n* = 8) after tumor cell inoculation. SiLNs that were injected with KM-Luc/GFP cells but not removed were used as control (G-C group, *n* = 5). **b** FM3A-Luc cells were injected into the SiLN (*n* = 15) (FM3A group). The SiLN was removed 3 days (G-D3 group, *n* = 4), or 7 days (G-D7 group, *n* = 6) after tumor cell inoculation. SiLNs that were injected with FM3A-Luc cells but not removed were used as control (G-C group, *n* = 5). **c-d** The lung, PALN and liver metastasis rate in the control and SiLN removal groups. **e-f** The ex vivo luciferase activity of the lung in the control and SiLN removal group

We established the mouse model via surgical removal of the tumor-bearing lymph node promoting tumor metastasis in distant organs. The SiLNs bearing tumor cells were surgically removed at 3 and 6 days (KM group) or 7 days (FM3A group) after inoculation, respectively. The mice were observed by the IVIS (Fig. 1a, b). Lung metastasis first appeared 9 days after inoculation in the KM control group (G-C) without the surgical removal of the tumor-bearing SiLN and the lung metastasis rate was 20%. At 9 days after inoculation, the lung metastasis rate in the SiLN removal group (G-D3 and G-D6) was 82.35%, which was significantly higher than in the control group (*P* = 0.021) (Fig. 1c). Similarly, in FM3A group, Fig. 1d shows the lung metastasis rate was 20% in control group. Lung metastasis first appeared 21 days after inoculation. At 21 days after inoculation, the lung metastasis rate in the SiLN removal group was 100%, which was significantly higher than in the control group (*P* = 0.004).

The PALN metastasis rate was 20 and 23.53% in the control and SiLN removal groups for KM group, respectively, and there was no statistical significance. The PALN metastasis rate was 20% in both control and SiLN removal groups for FM3A group. The liver metastasis rate was 5.9% in the SiLN removal KM group, and no liver metastasis was found in the KM and FM3A control groups (Fig. 1c, d). The luciferase activity of the lung ex vivo in the SiLN removal group was significantly higher than in the KM and FM3A control group (Fig. 1e, f).

### IHC staining analysis of luciferase in PALN, lung and liver

To confirm the IVIS results, we stained sections of PALN, lung and liver with and without SiLN removal in KM and FM3A group for tumor cells marker luciferase.

**Fig. 2:**
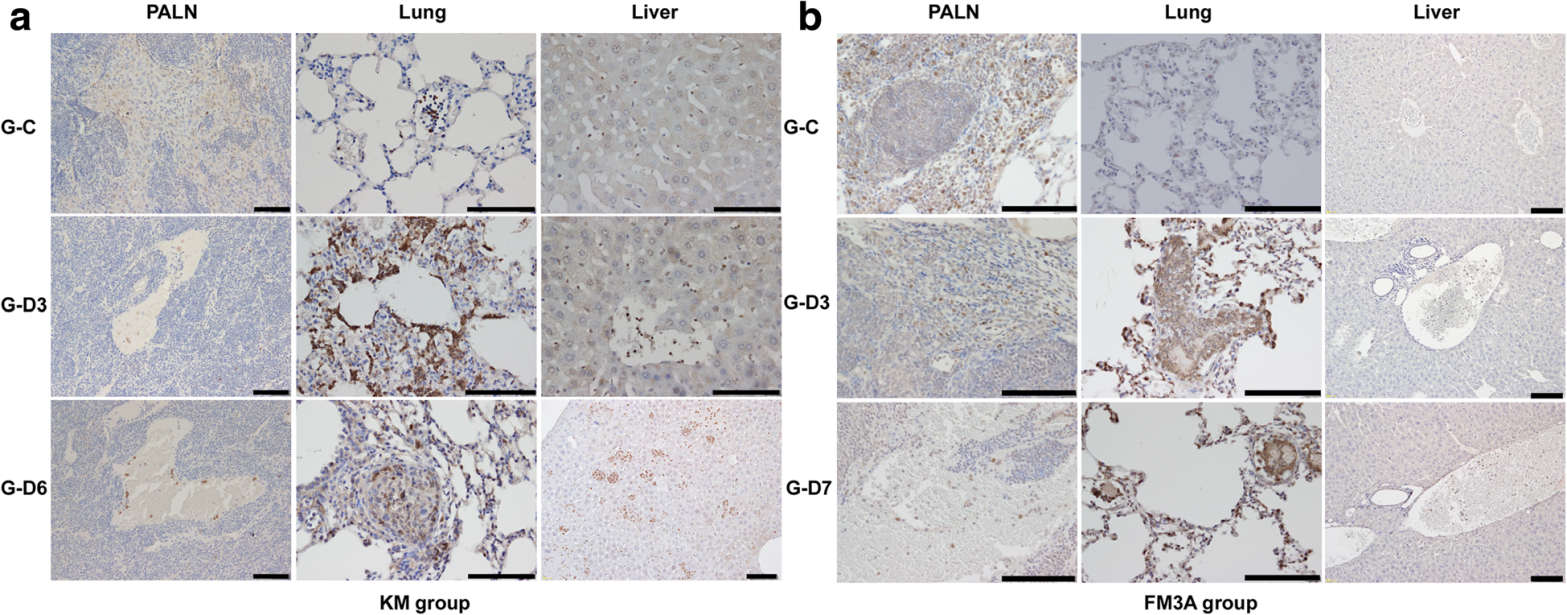
The expression of luciferase in PALN, Lung and liver. The Immunohistochemical (IHC) staining of luciferase in PLAN (left panel), lung (middle panel) and liver (right panel) of the KM (**a**) and FM3A (**b**) groups. Bar: 100 μm

In the KM and FM control groups, tumor nests were observed in some PALNs. In addition, scattered luciferase-positive tumor cells were seen in the blood vessels and between the alveolar epithelial cells in lung. At the same time, luciferase-positive tumor cells were seldom found in liver (Fig. 2a, b).

In both the KM and FM3A groups with the surgical removal of tumor-bearing SiLN, the IOD value of the luciferase-positive tumor cells in the PALNs were significantly lower than in the control groups (Table 1). Tumor nests of different sizes were observed around the terminal bronchi and in the distal alveoli in lung. Moreover, small satellite-like metastatic foci were observed in some livers and scattered luciferase-positive tumor cells were found within the liver blood sinus (Fig. 2a, b). Compared with the control group, the tumor burden in lung was significantly increased in all SiLN groups and there was statistical difference only between G-D6 and G-C of KM groups in liver (Table 1).

**Table 1:** Expression level of luciferase in PLAN, Lung and liver

		PALN		Lung		Liver	
		IOD Median(IQR)	*P* value	IOD Median(IQR)	*P* value	IOD Median(IQR)	*P* value
KM group	G-C	489.79(161.99)	0.001^a^	107.97(33.41)	< 0.001^a^	16.85(8.85)	0.254^a^
	G-D3	151.54(68.71)	0.945^b^	309.73(48.36)	0.005^b^	20.29(9.95)	0.106^b^
	G-D6	171.47(13.86)	0.002^c^	416.96(99.09)	< 0.001^c^	30.19(7.73)	0.003^c^
FM3A group	G-C	388.61(52.09)	0.016^a^	183.76(33.56)	< 0.001^a^	12.14(1.39)	0.421^a^
	G-D3	219.94(29.07)	0.009^d^	407.27(31.04)	0.127^d^	13.97(3.40)	0.691^d^
	G-D7	127.55(34.01)	0.004^c^	432.17(13.64)	0.002^c^	15.26(0.91)	0.310^c^

*P* < 0.05 was considered significant

*IOD* integral optical density

^a^compared with G-D3

^b^compared with G-D6

^c^compared with G-C

^d^compared with G-D7

### The lung metastatic niche

**Fig. 3:**
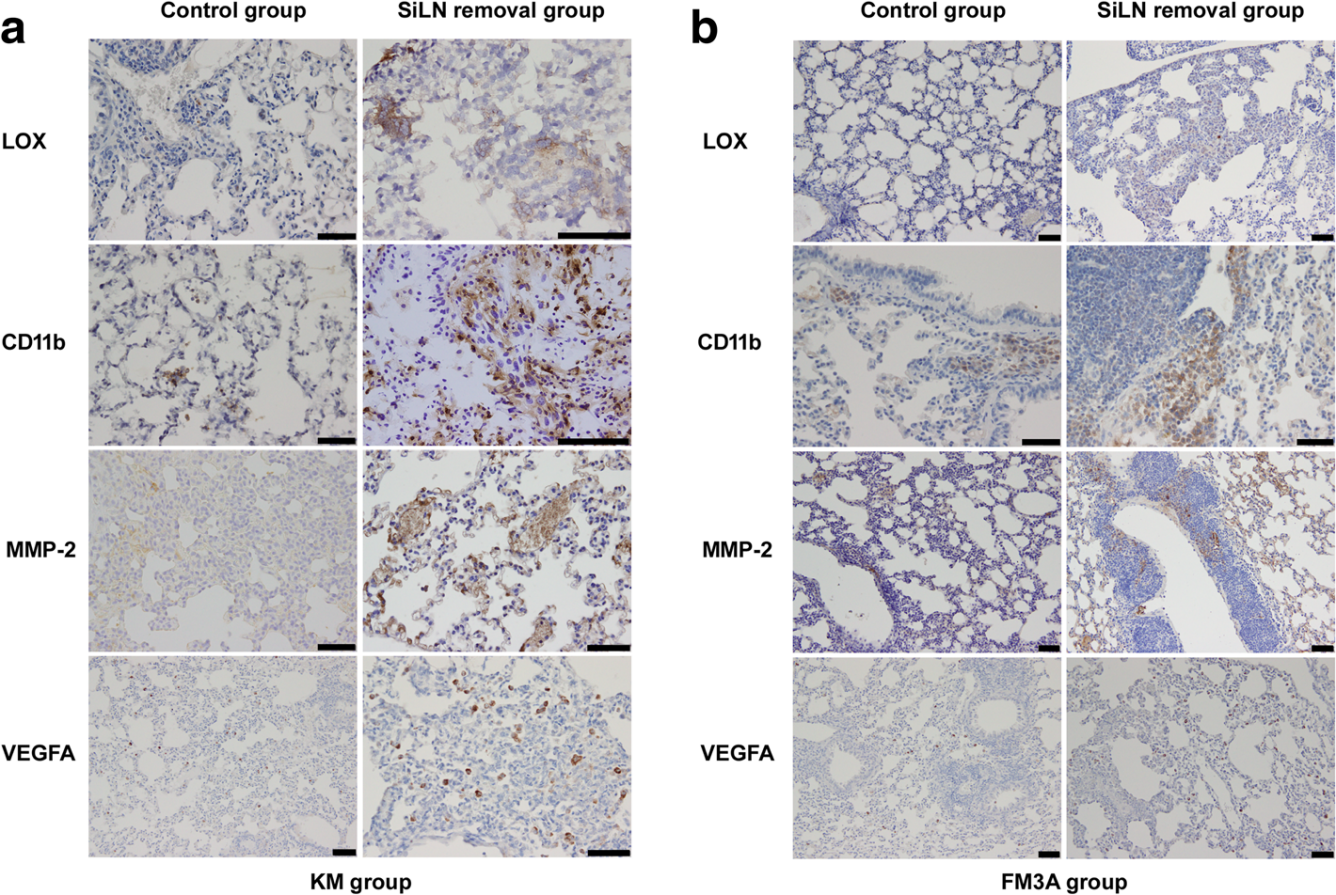
The changes of metastatic niche in lung samples. The expression of LOX, CD11b, MMP-2 and VEGFA were determined using IHC staining in the KM (**a**) and FM3A (**b**) groups. Bar: 50 μm

To investigate the influence of surgical removal of SiLN on metastatic site and explore the pathophysiology of the local tissue microenvironment in lung, we performed the immunohistochemical staining for LOX, CD11b, MMP-2, and VEGFA. LOX is critical for pre-metastatic niche formation and is essential for recruitment of BMDCs [19, 20]. LOX showed strong staining in both KM and FM3A SiLN removal groups.CD11b^+^ BMDCs have a variety of functions which may enhance metastatic tumor growth. We found CD11b^+^ clusters abundantly located in lung in KM and FM3A SiLN removal groups, while clusters of CD11b^+^ cells were seldom observed in both control groups. The expression of MMP-2 and VEGFA indicated a role in matrix changes and angiogenesis. The high expression of MMP-2 and VEGFA were also detected in our SiLN removal model (Fig. 3a, b).

Statistical analysis showed the expression of LOX, CD11b, MMP-2 and VEGFA in all of the KM and FM3A SiLN removal groups was significantly higher than in the corresponding control group (Table 2). We also analyzed the correlation among the LOX, CD11b, MMP-2 and luciferase by Spearman’s correlation analysis. In both the KM and FM3A SiLN removal groups, the expression level of LOX showed a positive correlation with the expression level of luciferase (*r* = 0.767, *P* = 0.001; *r* = 0.745, *P* = 0.013;Table 3) and CD11b (*r* = 0.733, *P* = 0.025; *r* = 0.721, *P* = 0.019; Table 3), respectively. The expression level of CD11b showed a positive correlation with the expression level of MMP-2 (*r* = 0.786, *P* = 0.036; *r* = 0.709, *P* = 0.022; Table 3).

**Table 2:** The expression level of LOX, CD11b, MMP-2 and VEGFA in lung

		KM group			FM3A group		
		Control	SiLN removal	*P* value	Control	SiLN removal	*P* value
IOD Median (IQR)	Lox	159.88(74.58)	432.19(133.55)	< 0.001	81.30(41.28)	527.83(72.49)	0.002
	CD11b	419.05(162.82)	1457.15(228.39)	< 0.001	480.03(58.28)	846.84(68.05)	< 0.001
	MMP-2	395.32(174.29)	1369.99(187.05)	< 0.001	327.07(63.52)	2098.03(165.68)	< 0.001
	VEGFA	107.46(24.58)	796.19(76.57)	0.002	82.35(15.72)	1192.30(279.41)	< 0.001

*P* < 0.05 was considered significant

*IOD* integral optical density

**Table 3:** Correlation among the expression level of LOX, luciferase, CD11b and MMP-2 in lung of KM and FM3A SiLN removal groups

		KM group	FM3A group
LOX	Luciferase	*P* = 0.001, r = 0.767	*P* = 0.013, r = 0.745
LOX	CD11b	*P* = 0.025, *r* = 0.733	*P* = 0.019, *r* = 0.721
CD11b	MMP-2	*P* = 0.036, *r* = 0.786	*P* = 0.022, *r* = 0.709

*P* < 0.05 was considered significant; r: Spearman correlation coefficient

### Matrix changes promotes metastatic tumor growth in the lung

**Fig. 4:**
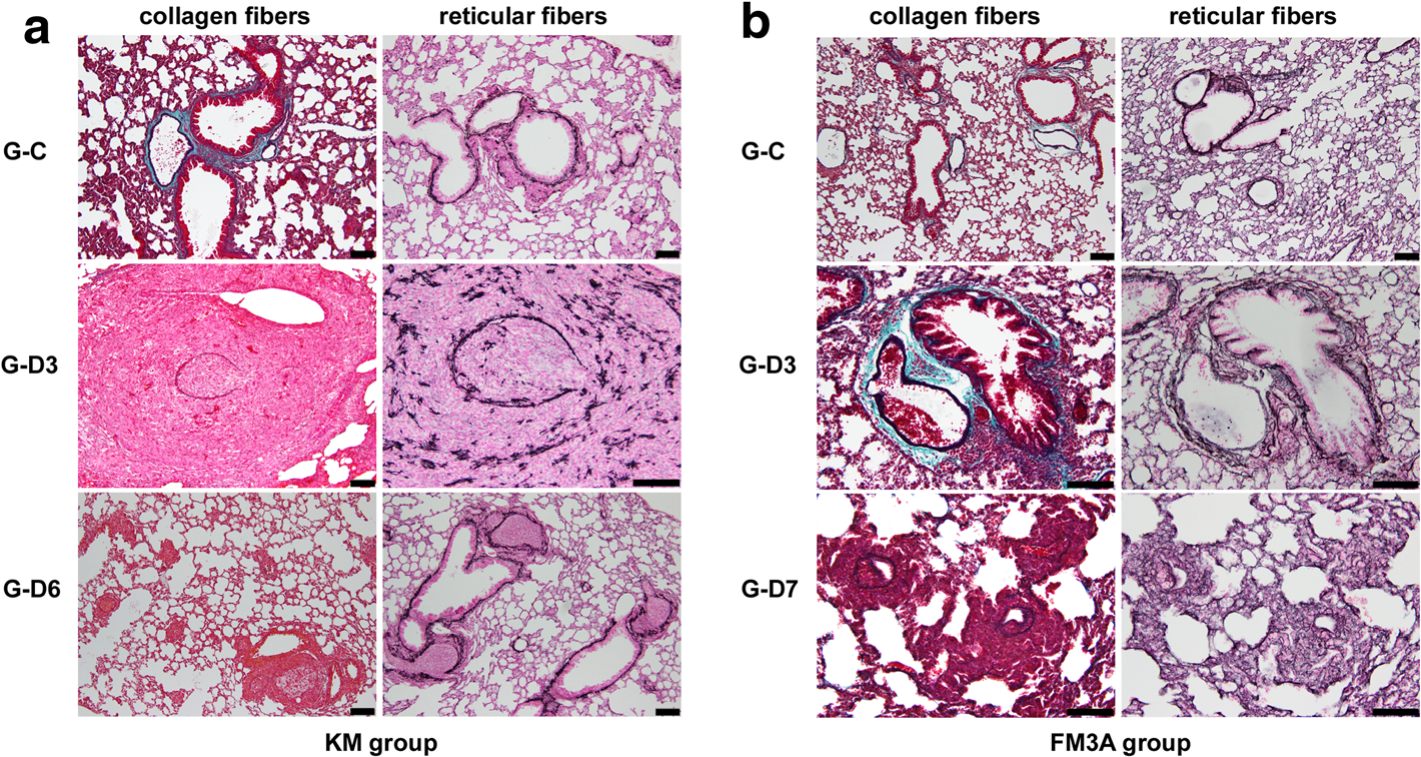
Location and expression of collagen fibers and reticular fibers. Location and expression of collagen fibers (left panel) and reticular fibers (right panel) were analyzed by Elastic-Masson (EM) staining and silver impregnation method in the KM (**a**) and FM3A (**b**) groups. Bar: 100 μm

MMP-2 is known for cleaving extracellular matrix. Using Elastic Masson staining and silver impregnation method, we observed the changes of collagen fibers and reticular fibers. With EM staining, blue-green collagen fibers were determined to be evenly located around the bronchi and blood vessels in the control group. Collagen fibers located around the bronchi and blood vessels in the SiLN removal groups were sparse and almost not found. Positive silver-stained reticular fibers were black. The reticular fibers in the lungs in both control groups were slender, loosely arranged, and interwoven into networks. Compared with the corresponding control group, the reticular fibers in the lungs in all of the SiLN removal groups were obviously increased and fractured (Fig. 4a, b).

The difference of collagen fiber per area was significant between the control group and the SiLN removal groups in both KM and FM3A groups. The difference of reticular fiber per area was significant between the control group and the SiLN removal groups in both KM and FM3A groups (Table 4). The expression level of MMP-2 showed a negative correlation with the collagen fibers per area (*r* = − 0.847, *P* = 0.016;*r* = − 0.748,*P* = 0.013;Table 5). Moreover, the per area of collagen fibers showed a negative correlation with the expression level of luciferase-positive tumor cells in both the KM and FM3A SiLN removal groups (*r* = − 0.865, *P* = 0.012;*r* = − 0.681, *P* = 0.030;Table 5), which mean that the quantity of collagen fibers around the bronchi and blood vessels were negatively correlated with the tumor metastatic foci burden.

**Table 4:** Per area of collagen fibers and reticular fiber in lung

		Collagen fibers		Reticular fibers	
		per area (%)	*P* value	per area (%)	*P* value
KM group	G-C	0.0148(0.0045)	0.008^a^	0.0118(0.0018)	0.001^a^
	G-D3	0.005(0.001)	0.464^b^	0.0209(0.0013)	0.008^b^
	G-D6	0.0043(0.0021)	0.002^c^	0.026(0.005)	0.008^c^
FM3A group	G-C	0.038(0.009)	0.001^a^	0.025(0.004)	0.001^a^
	G-D3	0.024(0.007)	0.006^d^	0.044(0.008)	0.019^d^
	G-D7	0.007(0.003)	0.003^c^	0.054(0.012)	0.002^c^

*P* < 0.05 was considered significant; Data are expressed as the median (IQR)

^a^compared with G-D3

^b^compared with G-D6

^c^compared with G-C

^d^compared with G-D7

**Table 5:** Correlation among per area of collagen fibers, the expression level of luciferase and MMP-2 in lung of KM and FM3A SiLN removal groups

		KM group	FM3A group
collagen fibers	MMP-2	*P* = 0.016, *r* = −0.847	*P* = 0.013, r = − 0.748
collagen fibers	Luciferase	*P* = 0.012, *r* = − 0.865	*P* = 0.030, *r* = − 0.681

*P* < 0.05 was considered significant; r: Spearman correlation coefficient

### Surgical removal of SiLN enhanced expression of LOX and CD11b in liver

**Fig. 5:**
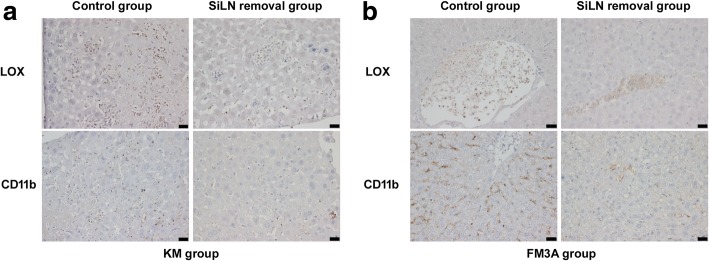
The changes of metastatic niche in liver samples. The expression of LOX and CD11b were observed using IHC staining in the KM (**a**) and FM3A (**b**) groups. Bar: 20 μm

We also investigated the expression of LOX and CD11b in the liver tissues (Fig. 5a, b). The situation was similar to the data obtained in the lungs. The expression levels of LOX and CD11b in the liver in all of the KM and FM3A SiLN removal groups were significantly higher than in the corresponding control group (*P* < 0.001, Table 6).

**Table 6:** The expression levels of LOX and CD11b in liver

		KM group			FM3A group		
		Control	SiLN removal	*P* value	Control	SiLN removal	*P* value
IOD Median(IQR)	Lox	146.78(17.19)	318.88(53.53)	< 0.001	102.35(30.71)	210.76(43.29)	< 0.001
	CD11b	376.03(50.23)	755.90(21.10)	< 0.001	319.62(33.35)	588.03(34.53)	< 0.001

IOD: integral optical density; *P* < 0.05 was considered significant

### Anti-tumor response in tumor-draining PALN

**Fig. 6:**
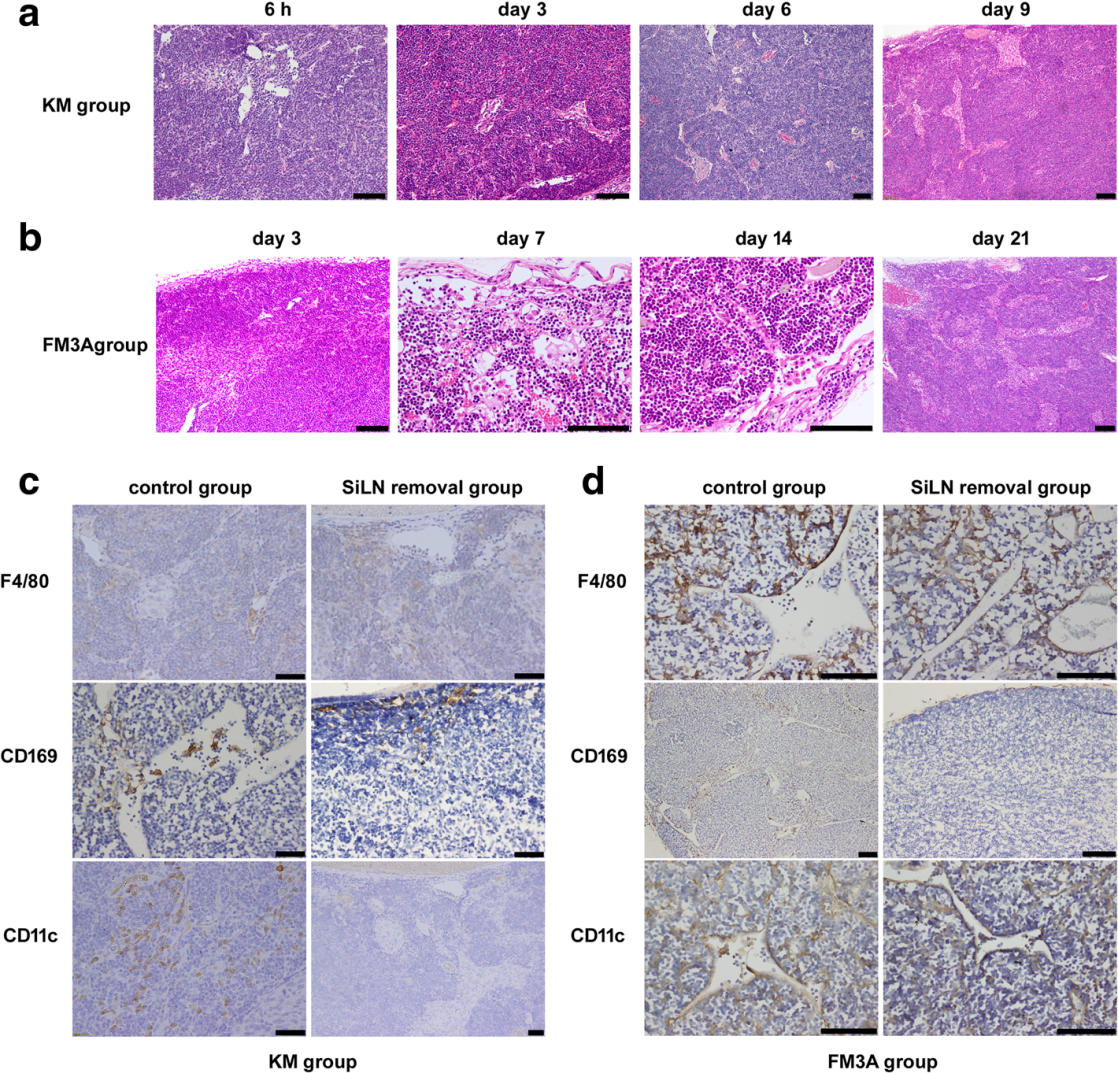
Histological changes in tumor-draining PALN. **a-b** HE staining of PALN in mice in KM (**a**) and FM3A (**b**) control groups. As time went by, tumor cells were found in lymphatic sinuses and tumor nests formed. **c-d** The expression of F4/80, CD169 and CD11c in PALN were analyzed by IHC staining. Bar: (**a-c**) 100 μm, (**d**) 50 μm

To observe morphological and histological changes in PALN, we performed HE staining. Tumor cells flowed into the subcapsular sinus of the PALN 3 and 7 days after inoculation in the KM and FM control groups, respectively. As time went by, tumor cells were found in lymphatic sinuses and tumor nests formed (Fig. 6a, b).

Lymph nodes play a key role in orchestrating immune response against tumors. An important event is the presentation of tumor antigens within the tumor-deriving lymph node. Macrophages and dendritic cells are professional antigen-presentation cells. Macrophages can be identified according to the expressions of F4/80 and CD169. We did not observe significant changes in the numbers of F4/80^+^ mature macrophages between KM and FM3A control groups and SiLN removal groups, respectively. However, we found that the numbers of CD11C^+^ and CD169^+^ cells in the PALNs in both the KM and FM SiLN control groups were significantly higher than in the corresponding SiLN removal groups (*P* < 0.001, Fig. 6c, d; Table 7).

**Table 7:** The expression levels of F4/80, CD169 and CD11c in tumor-draining PALN

		KM group			FM3A group		
		Control	SiLN removal	*P* value	Control	SiLN removal	*P* value
IOD Median (IQR)	F4/80	8431.42(2520.87)	7726.41(824.07)	0.423	5172.4(552.41)	5028.82(1072.5)	0.356
	CD169	3213.51(240.77)	1026.38(281.09)	< 0.001	1394.34(489.14)	622.89(254.42)	< 0.001
	CD11c	368.04(65.7)	179.87(80.47)	< 0.001	486.99(168.8)	173.73(74.75)	< 0.001

*P* < 0.05 was considered significant

*IOD* integral optical density

## Discussion

An operation to treat cancer includes resection of the primary lesion, lymphadenectomy, reconstruction, and so on. To date, surgery has been considered to be the primary therapeutic regimen for most malignancies, even though sometimes the effect of surgery is not ideal, since the surgical process could increase the risk of metastases of residual cancer cells to other organs [1, 4, 21]. Experimental data suggest that increased surgical stress augments cancer metastasis via surgical stress-induced expression of proteinases in the target organ of metastasis [22]. The effect of surgery on metastasis may be attributed to a number of factors, including immunosuppression after surgical stress, action of cytokines or changes of tumor microenvironment [23]. To explain the presence of postoperative tumor metastasis, it is necessary to find the appropriate in vivo model, thereby gaining an in-depth understanding of the mechanism of tumor removal-induced metastasis in order to develop effective treatment options and to improve metastatic cancer prevention and treatment.

Our IVIS results showed that the surgical removal of tumor-bearing SiLN promoted the incidence of lung metastasis (Fig. 1). Further, immunohistochemistry showed that luciferase-positive tumor cells were sparsely spread in the control lung tissues and was significantly lower than in the SiLN removal group. Although there was no significant difference in the rate of PALN metastasis between the control and tumor-bearing SiLN removal group, the number of luciferase-positive tumor cells in PALN in the surgical SiLN removal group was significantly less than the control group (Fig. 2, Table 1).

The pre-metastatic niche is defined as microenvironment with a series of molecular and cellular changes, which forms a supportive and receptive pre-metastatic site and the fertile “soil” to prepare for the colonization of “seed”, that is metastatic tumor cells, thereby facilitating tumor cell adhesion and growth in the distant organs and promoting tumor metastasis [8]. Studies have shown that local and high-precision radiotherapy does not induce direct injury, but it directly kills or stimulates the tissue cells compared with surgery, which also accelerates the incidence of tumor metastasis during anti-cancer therapy [2, 24]. Previous studies have shown that local radiotherapy causes a stronger expression of angiogenic factors than anti-angiogenic factors and disturbs the configuration imbalance of VEGFs in the vascular bed of the metastatic site, which then results in outbreak growth of dormant metastatic tumors [24, 25]. Adjuvant applications of the exogenous VEFGR inhibitor cediranib after radiotherapy restored tumor inhibition, and studies have shown that using angiogenesis inhibitors to target the VEGF pathway or knocking out the VEGF gene in mouse models with pancreatic cancer or glioblastoma has anti-cancer therapeutic effects by reducing the tumor volume and prolonging survival, but the two actions change tumor phenotypes and enhance tumor invasion and metastasis [26–28]. The discontinuation of VEGF inhibitors still enhances the tumor invasion, suggesting that this treatment increases the persistence of tumor invasion. This implies that not only does the treatment-induced trauma lead to tumor-promoting responses, but the disappearance of the tumor itself also promotes tumor metastasis during anti-cancer therapy. For the tumor treatment targeting a single angle, tumors can derive responses of increased invasiveness and extended metastasis. Therefore, a comprehensive consideration of all characteristics of the tumor microenvironment may accomplish a radical anti-cancer treatment.

Studies have shown that LOX secreted from hypoxic primary tumor cells accumulates in the pre-metastatic sites; LOX is an indispensable factor for recruiting bone marrow-derived CD11b^+^ cells (i.e., immature myeloid progenitor cells) to metastatic sites [19, 20]. LOX expression in SiLN removal groups was significantly higher than in the control group and was positively correlated with luciferase expression. These findings are consistent with the results of previous studies [19, 20], suggesting that high LOX expression in the metastatic sites was closely associated with the colonization and metastasis of tumor cells. Moreover, the number of CD11b^+^ cells in the lung and liver of the mice in the SiLN removal groups was significantly increased, which was positively correlated with LOX expressions (Fig. 3, Tables 2, 3).

Since the activation of MMP increased the invasion of BMDCs [19, 29], we detected MMP-2 expression in the lung tissues of the surgical SiLN removal groups, which was interestingly higher than that in the control groups and it was positively correlated with CD11b expression (Fig. 3, Tables 2, 3).

Our results showed that the degradation of the collagen fibers around the pulmonary vessels in the lungs of mice with the surgical removal of tumor-bearing SiLN was quite significant and the quantity of collagen fibers was negatively correlated with the expression of MMP-2, leading to a decrease in barrier function, thereby providing a channel for the tumor cells to invade blood vessels and establishing helpful conditions for tumor cell metastasis. The fracture of reticular fibers in the SiLN removal groups facilitated tumor cell invasion, thereby accelerating lung metastasis (Fig. 4, Table 5).

The above results suggested that high LOX and MMP-2 expressions and a great number of CD11b^+^ cell facilitate the formation of the metastatic niche and the colonization, growth, and invasion of tumor cells in the lung. The accumulation of LOX at the metastatic site promoted CD11b^+^ BMDC adhesion, MMP-2 production, and degradation of intra-pulmonary vascular collagen. Moreover, the morphological changes of the extracellular matrix scaffold remodeled the local microenvironment and enhanced tumor cell invasion. Previous studies have shown that BMDC induced interstitial epithelial transformation of the disseminated tumor cells [30], secreted chemokines to enhance metastasis and nesting of tumor cells [31], and lowered in vivo immune surveillance by immunosuppression [7, 8, 32]. The current study also showed that VEGFA expression in the surgical removal groups was significantly higher than in the control group, and it is known that the secretion of VEGF and other angiogenic factors promote vascular angiogenesis, thereby possibly playing a synergistic role in promoting tumor metastasis.

The current study also identified LOX, CD11b, and luciferase expression in the liver and showed similar expression patterns of LOX and CD11b in the lung (Fig. 5). Interestingly, although the ex vivo IVIS results showed that the liver metastasis was only 5.9% in KM SiLN removal group, immunohistochemical staining showed that luciferase-positive tumor cells were scattered in liver sinusoids in all of the SiLN removal groups (Fig. 2), which might be associated with tumor cell types and organotropic targeting [33], unfavorable niche formation, or the short observation time in this study. Further studies will be necessary to address the above concerns.

Lymph nodes are the well-known routes for lymphatic metastasis and the primary place for anti-tumor immune responses. The current study observed the influx of tumor cells into the lymphoid sinus of PALN in the control group, which resulted in tumor nest formation, the retention of tumor cells, and the reduction of tumor cell metastasis to distant organs over time (Fig. 6). A study by Asano et al. [34] showed that subcutaneously injected tumor cells can be transported to lymph nodes (LNs) through the lymphatic flow and were phagocytosed by CD169^+^ macrophages in the LN subcapsular sinus, which then directly cross-present the dead cell-associated antigens to CD8^+^T cells. The current study found a large luciferase-positive cell influx into the subcapsular sinus in the PALN of the control group. In addition, the number of CD169^+^ and CD11c^+^ cells in the control group was significantly higher than in the SiLN removal groups (Fig. 6, Table 7), suggesting the production of anti-tumor immune responses, which, to a certain extent, reduced the chance of tumor cell metastasis to the distant organs, such as the lung and liver.

There is growing data to support that noncoding RNAs (ncRNAs) play a significant role in tumor metastasis [35, 36]. A diversity of ncRNAs was demonstrated to promote proliferation and metastasis of tumor cells [37–39]. We preformed additional microarray detection and indicated expression differences in both miRNAs and lncRNAs (data not shown) to clarify the specific mechanism associated with our findings. Our study will further demonstrate certain miRNAs and lncRNAs could be pre-metastatic biomarkers, prognostic tools and potential therapeutic targets.

## Conclusions

Our mouse model with the surgical removal of the tumor-bearing lymph node greatly simulated the incidence of tumor metastasis after surgery in clinical practice [4, 21] and allowed us to observe metastatic niche changes in distant organs. Our study indicates that surgical removal of the tumor-bearing lymph node promoted tumor metastasis to lung and liver; higher expression level of LOX and a larger number of CD11b^+^ cells in lung and liver facilitated the recruitment and colonization of disseminated tumor cells; higher CD169^+^ and CD11c^+^ cells number in PALN in groups without surgical removal of SILN might reduce the tumor metastasis to lung and liver.

A greater understanding of the formation of the metastatic niche will help our investigation into the prevention and treatment of tumor metastasis.

## Abbreviations

BMDCs: Bone marrow-derived cells
DAB: Diaminobenzidine
EM staining: Elastic-Masson staining
IOD: Integral optical density
LNs: Lymph nodes
LOX: Lysine oxidase
MMP-9: Matrix metalloproteinase − 9
MXH10/Mo/lpr mice: MXH10/Mo-*lpr*/*lpr* mice
ncRNAs: Noncoding RNAs
PALN: Proper axillary lymph node
PBS: Phosphate-buffered saline
RT: Room temperature
SiLN: Subiliac lymph node
TDSFs: Tumor-derived secreted factors
VEGF: Vascular endothelial growth factor
